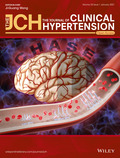# Featured Cover

**DOI:** 10.1111/jch.14175

**Published:** 2021-01-19

**Authors:** Jingjing Zhao, Fang Yuan, Feng Fu, Yi Liu, Changhu Xue, Kangjun Wang, Xiangjun Yuan, Dingan Li, Qiuwu Liu, Wei Zhang, Yi Jia, Jianbo He, Jun Zhou, Xiaocheng Wang, Hua Lv, Kang Huo, Zhuanhui Li, Bei Zhang, Chengkai Wang, Li Li, Hongzeng Li, Fang Yang, Wen Jiang, the CHASE Study Group

## Abstract

The cover image is based on the Original Paper *Blood pressure variability and outcome in acute severe stroke: A post hoc analysis of CHASE—A randomized controlled trial* by Jingjing Zhao et al., https://doi.org/10.1111/jch.14090.